# RelB and RelE of *Escherichia coli* Form a Tight Complex That Represses Transcription via the Ribbon–Helix–Helix Motif in RelB

**DOI:** 10.1016/j.jmb.2009.09.006

**Published:** 2009-11-27

**Authors:** Martin Overgaard, Jonas Borch, Kenn Gerdes

**Affiliations:** 1 Department of Biochemistry and Molecular Biology, University of Southern Denmark Odense, Campusvej 55, 5230 Odense M, Denmark; 2 Centre for Bacterial Cell Biology, Institute for Cell and Molecular Biosciences, Medical School, Newcastle University, Newcastle NE2 4HH, UK

**Keywords:** RHH, ribbon–helix–helix, TA, toxin–antitoxin, WT, wild type, SPR, surface plasmon resonance, HMK, heart myosin kinase, X-gal, 5-bromo-4-chloro-3-indoyl-β-d-galactoside, EDTA, ethylenediaminetetraacetic acid, toxin, antitoxin, RelB, RelE, ribbon–helix–helix

## Abstract

RelB, the ribbon–helix–helix (RHH) repressor encoded by the *relBE* toxin–antitoxin locus of *Escherichia coli*, interacts with RelE and thereby counteracts the mRNA cleavage activity of RelE. In addition, RelB dimers repress the strong *relBE* promoter and this repression by RelB is enhanced by RelE; that is, RelE functions as a transcriptional co-repressor. RelB is a Lon protease substrate, and Lon is required both for activation of *relBE* transcription and for activation of the mRNA cleavage activity of RelE. Here we characterize the molecular interactions important for transcriptional control of the *relBE* model operon. Using an *in vivo* screen for *relB* mutants, we identified multiple nucleotide changes that map to important amino acid positions within the DNA-binding domain formed by the N-terminal RHH motif of RelB. Analysis of DNA binding of a subset of these mutant RHH proteins by gel-shift assays, transcriptional fusion assays and a structure model of RelB–DNA revealed amino acid residues making crucial DNA–backbone contacts within the operator (*relO*) DNA. Mutational and footprinting analyses of *relO* showed that RelB dimers bind on the same face of the DNA helix and that the RHH motif recognizes four 6-bp repeats within the bipartite binding site. The spacing between each half-site was found to be essential for cooperative interactions between adjacently bound RelB dimers stabilized by the co-repressor RelE. Kinetic and stoichiometric measurements of the interaction between RelB and RelE confirmed that the proteins form a high-affinity complex with a 2:1 stoichiometry. Lon degraded RelB *in vitro* and degradation was inhibited by RelE, consistent with the proposal that RelE protects RelB from proteolysis by Lon *in vivo*.

## Introduction

Toxin–antitoxin (TA) loci are abundant gene cassettes present, often in multiple copies, on plasmids and chromosomes of Bacteria and Archaea.^1^ TA loci encode a stable “toxin” that inhibits translation or replication and an unstable antitoxin that neutralizes the toxin. Ectopic production of the toxins is highly inhibitory to cell growth, but the antitoxins form complexes with the toxins and thereby counterbalance toxin activity. Activation of TA loci depends on cellular proteases such as Lon or Clp that degrade the antitoxins.^2,3^

Two major classes of toxins have been identified based on their cellular targets. One class of toxins inhibits translation by mRNA cleavage (i.e., RelE and MazF) and is commonly referred to as mRNA interferases.^4–8^ Another class of toxins inhibits DNA gyrase and thereby inhibits replication (i.e., CcdB and ParE).^9–11^ The antitoxins are small modular proteins that contain an N-terminal structured DNA-binding domain, required for autoregulation of TA operon transcription, and a flexible C-terminus, required for antitoxin activity.^12–15^ Upon interaction with the cognate toxin, the C-terminus of the antitoxin becomes structured and results in the formation of a tight nontoxic protein complex.^16–18^ In almost all cases known, the TA complex binds to one or more operators in the TA promoter region and represses transcription of the TA operon.^19–21^ In its free state, the antitoxin is degraded by one or more cellular proteases.^2,3,22–26^ Thus, the cellular levels and turnover rates of the proteins are adjusted by a combination of autoregulation and proteolysis. Usually, the TA genes are translationally coupled and produce antitoxin in excess of the toxin, ensuring efficient inactivation of the latter,^27, 28, 29^ although exceptions to this paradigm probably exist.^8^

The *relBE* locus of *Escherichia coli* encodes mRNA interferase RelE that cleaves mRNA positioned at the ribosomal A-site and antitoxin RelB that counteracts this activity.^5,30^ RelB copurifies with RelE and the proteins interact in the yeast two-hybrid system.^31,32^ The *relBE* operon is autoregulated by RelB, which alone functions as a repressor of transcription. The RelBE complex represses transcription more efficiently than RelB alone; thus, RelE functions as a co-repressor of transcription.^19,33,34^ During steady-state cell growth, *relBE* transcription is efficiently repressed due to autoregulation by the RelBE complex.^2,19,33^ By contrast, conditions that inhibit translation, such as amino acid starvation, induce *relBE* transcription and concomitantly activate RelE.^2,19,35,36^ The metabolic turnover of RelB depends on Lon protease and degradation of RelB was suggested to explain the strongly increased *relBE* transcription during amino acid starvation.^2^ Recently, we showed that RelB and RelE form a tight RelB_2_·RelE complex that bound cooperatively to the *relO* operator in the *relBE* promoter region.^33^ Interestingly, *relBE* transcription was controlled by the RelB/RelE ratio rather than the absolute amounts of the proteins. Thus, with excess RelB, RelE strongly enhanced binding of RelB to the operator and repressed transcription. By contrast, excess RelE prevented RelB binding to *relO in vitro* and stimulated *relBE* operon transcription *in vivo*. Thus, excess RelE triggered *relBE* transcription.

The solution structure of a RelB dimer was obtained recently.^34^ Consistent with our findings,^33^ this study showed that a RelB dimer recognizes a hexad repeat in the palindromic operator through an N-terminal ribbon–helix–helix (RHH) motif and that RelE enhances the affinity of adjacent bound RelB dimers for the operator element. Moreover, it was demonstrated that the flexible C-terminus of RelB is required for RelB dimers to dimerize.

To gain further insight into the molecular interactions controlling the *relBE* transcription and RelE activity, we have undertaken a genetic and biochemical study of the regulatory properties of RelB. Using an *in vivo* screen for *relB* mutants defective in autoregulation, we identify amino acid residues within the RHH motif of RelB important for DNA binding. By mutational analysis of *relO* and hydroxyl radical footprinting, we show that RelB occupies four hexad repeats within *relO* with the core sequence [A/T]TGT[A/C]A. By nucleotide insertions, we show that no spacing is allowed between each of the two *relO* half-sites in order for the repression complex to maintain autoregulation. The appearance of free RelE is prevented by tight subnanomolar interaction to RelB and an ∼ 10-fold lower *in vivo* concentration to that of its cognate antitoxin. The ATP-dependent Lon protease binds to RelB and stimulates its degradation. Together, these results provide a quantitative and mechanistic basis for how the activity of the model RelE mRNA interferase is controlled.

## Results

### Random mutagenesis of *relB*

**Fig. 1:**
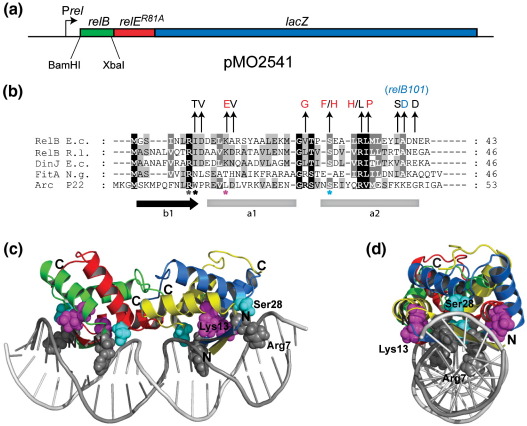
RelB mutants defective in transcriptional autoregulation. (a) Schematic of pMO2541 carrying a translational *relBE^R81A^::lacZ* fusion used to insert mutagenic PCR fragments of *relB* in order to screen for derepressed (blue) mutant colonies on X-gal indicator plates. (b) Multiple alignment of the RHH motifs of RelB, *E. coli*; RelB, *R. leguminosarum*; RelB2 (DinJ), *E. coli*; FitA, *N. gonorrhoeae*; and Arc, bacteriophage P22. Residues are marked by decreasing conservation: white text on black boxes; white text on dark grey boxes and black text on grey boxes. Vertical arrows indicate the amino acid substitutions that were found in the RelB RHH variants obtained in the screen. Amino acids shown in black were obtained in combination with one of the other mutations, whereas amino acids colored in red were obtained independently in each clone. The aspartate colored in blue is encoded by the previously described *relB101* allele. Amino acids in the alignment marked by asterisks were selected for further mutant characterization in Fig. 2 and are color coded according to representations in (c) and (d). The location of the RHH secondary structure in RelB is depicted by a black arrow and grey boxes, respectively. (c) Side view of a structure model of the RelB–DNA complex based on the NMR structure of RelB_1–50_ (PDB ID 2k29) and the crystal structure of the Arc–DNA complex (PDB ID 1bdt). Two RelB_1–50_ dimers were modeled on a 22-nucleotide double-stranded fragment representing Arc operator DNA. RelB_1–50_ monomers of each dimer are colored green and red or yellow and blue, respectively. Side chains of the conserved R7 amino acid (grey spheres) from the β-strand of each subunit make sequence-specific base interactions in DNA major grooves. Side chains of the conserved S28 (cyan spheres) from the N-terminal part of helix α2 make unspecific DNA sugar–phosphate backbone interactions. Side chains of K13 (magenta spheres) from the N-terminal part of helix α1 make putative electrostatic DNA sugar–phosphate backbone interactions. (d) Side view of (c) by 90° rotation along the *y*-axis. The representations were prepared in PyMOL.^37^

To identify amino acid residues in RelB important for autoregulation of the *relBE* operon, we constructed an *in vivo* screen based on a plasmid (pMO2541) in which the *relBE*^*R81A*^ operon was fused in-frame to *lacZ* (Fig. 1a); *relE*^*R81A*^ encodes a nontoxic version of RelE.^31^ In keeping with our previous finding that RelB autoregulates *relBE* transcription,^19,33^ this plasmid (pMO2541) expressed a very low level of LacZ activity in a Δ*relBE* deletion strain (< 1 U, data not shown). Since RelE is required for efficient autorepression, this low level of LacZ activity indicated that the LacZ portion of the RelE::LacZ fusion protein did not interfere with the co-repressor function of RelE.

Using silent mutations, we engineered two restriction sites into either end of *relB*, thus being able to insert *relB*-encoding DNA fragments that had been mutagenized by PCR into the natural context of *relB* (codons 4–76). Next, we screened for mutant alleles yielding dark blue colonies on X-gal (5-bromo-4-chloro-3-indoyl-β-d-galactoside) indicator plates. As expected, most such mutations were located in the RHH motif of RelB (Fig. 1b, vertical arrows). Some mutations (base transversions or transitions) were found together with one of the other mutations (Fig. 1b, residues colored black), while most mutant alleles (residues colored red) only contained single base mutations. We did not observe any mutations leading to substitutions in the conserved signature residue R7 of RelB; however, mutations were found encoding residues specific for the RelB family (I8, D9 and K13) (Fig. 1b) and also in the conserved residues S28, R32 and L33. Interestingly, we also obtained the *relB101* allele A39D that leads to “hyperactivation” of RelE due to metabolic instability of RelB^A39D^ (Ref. 23). The *relB101* mutation was originally found in a genetic screen that identified mutations that rendered the cell unable to inhibit synthesis of stable RNA during amino acid starvation,^38^ the so-called delayed relaxed response.

To map our mutations on the RelB structure, we constructed a model of (RelB_2_)_2_–DNA based on the previous published NMR structure of the RelB N-terminal domain,^34^ the Arc–DNA crystal structure,^39,40,41^ and our previous published stoichiometric data^33^ (Fig. 1c and d). In the model, two important sets of protein–DNA contacts are observed: β-sheets that insert into the DNA major grooves making crucial sequence-specific nucleotide base contacts (S3, D5 and R7) and protein–DNA backbone contacts involving amino acids in the N-terminus of the second α-helix α2 (S28). We obtained mutations in S28 (S28F and S28H) that abolished autoregulation, which could indicate that protein backbone amide groups in combination with side-chain specific hydrogen bonds make crucial contacts with the DNA phosphate groups on either side of the major grooves (Fig. 1c and d). In addition, we obtained K13E substitutions. Lysine 13 is a surface-exposed basic residue that potentially can form additional nonspecific contact through electrostatic interaction to the DNA backbone phosphate on both sides of the DNA helix (Fig. 1d). This interaction might, in combination with the contacts made by S28, guide or anchor the RelB dimers for correct positioning and sequence-specific nucleotide base contacts between the antiparallel β-sheets of RelB and the DNA major grooves in the operator region. Yet other mutations in *relB* yielded amino acid changes in key residues of the hydrophobic core in the RHH domain that probably do not contribute directly to protein–DNA contacts (i.e., I8, L33 and I38). Another substitution, which maps to the α1–α2 loop (V25G), could potentially affect DNA binding by changing the relative position of α1 to α2 crucial for anchoring DNA backbone contacts by the N- terminus of helix α2.

### DNA-binding properties of RelB with amino acid changes in the RHH motif

**Fig. 2:**
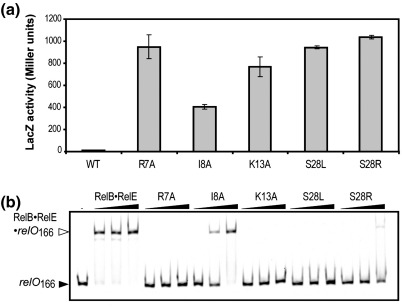
*In vivo* and *in vitro* characterization of amino acid changes in the RHH motif of RelB. (a) Autoregulation of *relBE* RHH mutants. Strains carrying plasmid derivatives of the low-copy-number R1 transcriptional fusion *relBE^R81A^::lacZ* vector, pMGJ4004, with the *relB* mutations indicated, were grown exponentially in LB medium and subjected to β-galactosidase assay. Each value represents the mean of at least three independent experiments. Error bars represent standard deviation of the mean. (b) Gel-shift analysis of RelB_2_·RelE repression complexes carrying substitutions in the DNA-binding RHH domain of RelB. A Cy5-labeled PCR fragment (*relO*_166_) was incubated in the absence (−) or presence of increasing concentrations (0.05, 0.25 and 1.25 μM) of either WT RelB_2_·RelE complex or the mutant derivatives as indicated and subjected to native PAGE and subsequent fluorescent scanning.

The above-described amino acid changes in RelB in all cases abolished autoregulation of the *relBE* operon. To gain direct evidence for defects in *relO* operator binding, we analyzed the autoregulatory and DNA-binding properties of RelB RHH variants R7A, I8A, K13A and S28L/R (Fig. 1b, asterisks). In the first three RelB variants, we introduced a single alanine substitution such that the properties of specific residues could be compared. For S28 we chose to create two substitutions (S28L, S28R). In the dimeric Arc repressor, homologous replacements (S35L, S35R) disrupt cooperative DNA binding^41^ (see the Discussion). The transcriptional activity of the *relBE* promoter was measured using a series of *relBE^R81A^-lacZ* transcriptional fusion constructs based in an R1 low-copy-number transcriptional fusion plasmid (Fig. 2a). As in the genetic screening, the RelB RHH variants were coexpressed with nontoxic RelE^R81A^ encoded in cis (Fig. 2a). While wild-type (WT) RelB efficiently repressed the promoter to below 10 U, the RHH mutations R7A, S28L and S28R all resulted in more than 100-fold derepression. The I8A and K13A mutants resulted in 43- and 83-fold derepression, respectively (Fig. 2a).

Next, we measured DNA binding of the RelB RHH variants in complex with the co-repressor His_6_-RelE (RelE) in a gel-shift assay (Fig. 2b). The antitoxin variants copurified with RelE and behaved similar to WT RelB_2_·RelE with respect to tight interaction with the toxin (see also Fig. 5). In the gel-shift assay, a ternary complex of RelB_2_·RelE·*relO*_166_ formed at the lowest concentration (0.05 μM) of protein (Fig. 2b). No ternary complexes were observed for the RHH variants R7A, K13A and S28L, not even at the highest concentration of the protein complex (1.25 μM). Replacing I8 with an alanine lowered the affinity of the RelB_2_·RelE complex by ∼ 10- to 20-fold. For the S28R variant, a faint ternary complex was only visible at the highest concentration used (1.25 μM). The oligomeric states of WT RelB and the mutated variants were further compared using chemical cross-linking with BS^3^ [bis(sulfosuccinimidyl) suberate] (Fig. S1). All RHH mutant proteins exhibited oligomerization patterns similar to that of WT RelB. The most predominant bands corresponded to dimers and tetramers. At lower RelB concentrations, only the dimeric band was observed (data not shown). Interestingly, in the homologous dimeric Arc repressor, S35 (S28 in RelB) is located in the dimer–dimer interface and replacement to residues with larger side chains (e.g., arginine or leucine) disrupts cooperative binding.^41^ In summary, our DNA-binding assays, structural model and cross-linking data indicated that S28 is involved in DNA–backbone interactions and is not required for tetramerization of RelB.

### Scanning mutagenesis of *relO*

Previously, RelB_2_ and the RelB_2_·RelE complex were found to protect a region within P_*relBE*_ termed *relO* spanning the region − 10 to + 17 with respect to the transcription start site.^33^ We confirmed that the left and right boundaries of the operator were located around − 10 and + 20 by 5′ and 3′ promoter–operator DNA deletion analysis using gel-shift assays (Fig. S2a and b).

**Fig. 3:**
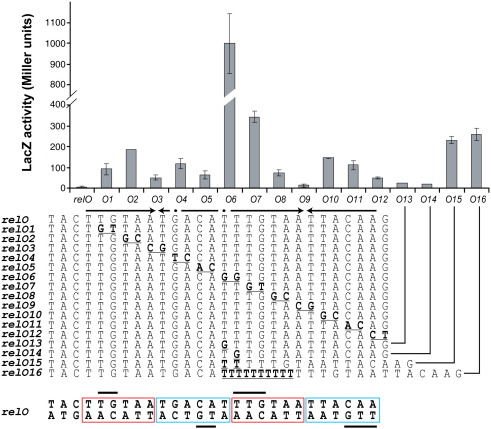
Scanning mutagenesis of *relO.* A series of dinucleotide substitutions (*relO1* to *relO12*), single base substitutions (*relO13* and *relO14*) and insertions (*relO15* and *relO16*) were introduced into pMGJ4004 and the resulting plasmids were transformed into strain MG1 (Δ*relBEF*::*aphA*) and cultures were grown exponentially in LB medium and subjected to β-galactosidase assay. Each value represents the mean of at least three independent experiments normalized to the LacZ β-galactosidase activity of the unrepressed mutant promoter relative to the corresponding WT promoter. Error bars represent standard deviation of the mean. A double-stranded representation of *relO* is given at the bottom of the figure. The hexad repeats of each operator half-site are boxed in red and blue, respectively, and protected positions obtained in Fig. 4 are marked by black bars.

To further define the sequence specificity required for high-affinity binding of RelB_2_·RelE to *relO*, we systematically introduced dinucleotide transversion substitutions in the promoter–operator region from − 10 to + 16 (Fig. 3, *relO1* through *relO14*). These mutations were introduced into R1 *lacZ* transcriptional fusion vectors containing either the entire *relBE*^*R81A*^ operon (pMGJ4004, repressed promoter) or only the *relB* promoter as a control for promoter activity (pKG4001, unrepressed promoter). Thus, the control plasmid was used to assess the effect of the mutations on the activity of the unrepressed *relBE* promoter (data not shown). The transcriptional activity of each promoter construct was measured during exponential cell growth. Mutations between positions − 10 and − 7 (TA and CT introduced into the − 10 box) abolished P_*relBE*_ promoter activity of the control plasmid and excluded them from the analysis. For the remaining constructs, the transcriptional activities of the repressed promoters (due to the presence of *relBE*^R81A^) were normalized with the activity of the unrepressed promoter (see Materials and Methods). Full repression of P_*relBE*_ was defined as the LacZ activity expressed from a plasmid (pMGJ4001) carrying the WT operator, *relO* (5.2 ± 2.4 U). Mutations on both sides of the pseudo palindromic half-site (TTGTAATGACAT) TG→GT, TA→GC (*relO1* and *relO2*) and GA→TC, CA→AC (mutants *relO4* and *relO5* in Fig. 3) resulted in significant derepression (12- and 36-fold, respectively), whereas the central AT (*relO3*) appeared less critical for repression. A similar pattern was observed for the perfect palindromic right half-site TTGTAATTACAA, with the exception of the first TG dinucleotide (*relO7*), which appeared to be more important than any other dinucleotide pair. Interestingly, substitution of the central TT pair resulted in a 190-fold derepression of transcription that may reflect the loss of RelB_2_·RelE binding to both half-sites of *relO* (*relO6*). To determine the contribution of each of the central T's, we extended the analysis and introduced single base transversions (*relO13* and *relO14*). In these cases, the promoter was derepressed 5- and 4-fold, respectively, indicating that the boundary between the two half-sites plays a pivotal role in maintaining cooperativity between adjacent bound RelB_2_·RelE complexes, possibly involving DNA bending similar as shown for the Arc repressor–DNA complex (Fig. 1c and d).

The above-described and previous findings showed that both half-sites of *relO* were required for cooperative binding of the RelB_2_·RelE complexes.^33,34^ Therefore, we introduced spacing between the two half-sites. Addition of only two nucleotides (*relO15*) that brought the two half-sites out of phase resulted in 45-fold derepression of *relBE* transcription. Insertion of eight additional T nucleotides (*relO16*) brought the half-sites back into phase; however, it failed to restore repression (50-fold derepression). Thus, the architecture of the RelB_2_·RelE complex bound to operator DNA requires both half-sites in close proximity. The mutational analysis of *relO* defined a hexad binding motif [A/T]TGT[A/C]A, repeated twice on each strand (Fig. 3, bottom), with the less critical nucleotide being the terminal A nucleotide of each subsite.

### RelB occupies four distinct subsites in *relO*

**Fig. 4:**
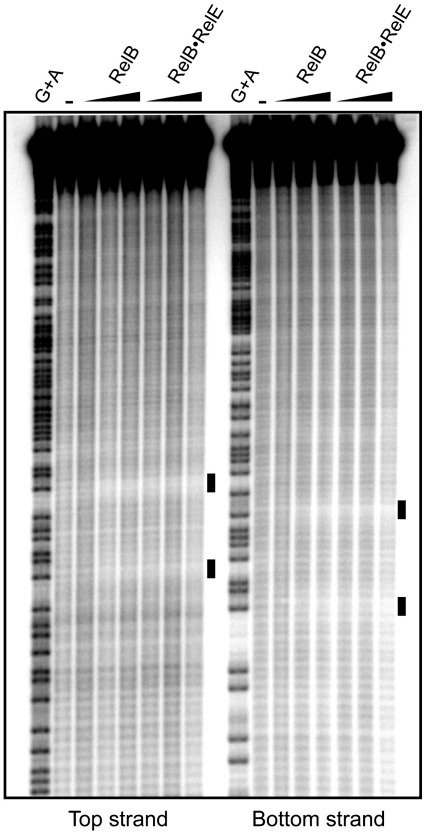
Hydroxyl radical footprinting of *relO*_166_. PCR fragments of *relO*_166_, ^32^P-end-labeled at the top and bottom strands, respectively, were incubated in the absence (−) or presence of increasing concentrations (0.1, 0.5 and 2.5 μM) of RelB_2_ or RelB_2_·RelE complex and subjected to hydroxyl radical footprinting. A ladder of G+A of each DNA fragment was included next to the footprinting reactions. Black bars indicate DNA sugar–backbone protected positions along *relO*, which are also indicated in Fig. 3.

Next, we performed hydroxyl radical footprinting on both DNA strands of *relO* using increasing concentrations of RelB and RelB_2_·RelE complexes (Fig. 4). Two discrete but distinct regions of protection (marked with filled bars) were observed for the two highest concentrations of RelB and for all three concentrations of the RelB_2_·RelE complex, confirming that RelB interacts directly with *relO* and that RelE acts to increase its affinity. This pattern was evident for both strands (Fig. 4, left and right). However, closer examination of these regions on both strands revealed that DNA backbone protection occurs at two base positions, T and G in one of the protected regions and at three base positions T, T and G in the other region. The strongest protection occurred at the backbone of the guanine base. These distinct sites confirmed the subtle asymmetry of the two half-sites and further define a *relO* core sequence of [a/t]TGt[a/c]a (Fig. 3). More importantly, the protected regions on each strand were interrupted by 9 and 10 bp, respectively, without any protection. This observation indicated that *relO* was occupied by two RelB dimers on the same face of the DNA, thus supporting the model shown in Fig. 1c and d.

### RelB forms a tight 2:1 heterotrimeric complex with RelE

**Fig. 5:**
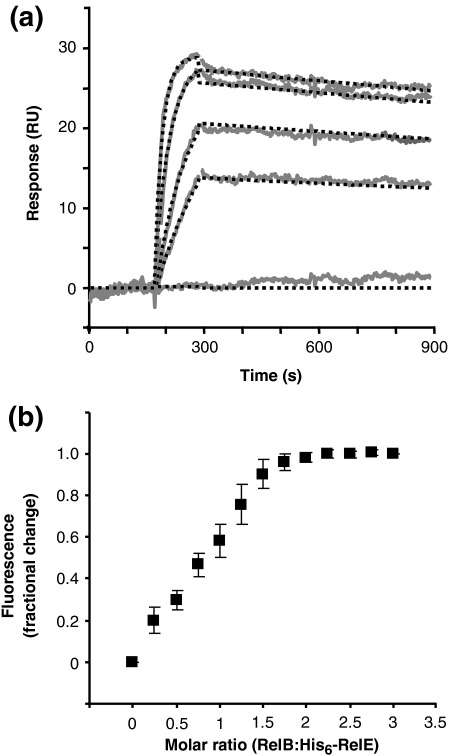
Kinetic and stoichiometric measurements of RelB and RelE interactions. (a) SPR kinetic analysis. Forty resonance units (RU) of RelB-Cys was immobilized on a CM5 sensor chip and assayed for concentration-dependent RelE binding. Increasing concentrations of RelE were injected in separate association/dissociation/regeneration cycles performed in duplicate for each concentration used. The reference-subtracted response difference in resonance units (RU) for each concentration used is shown in the sensorgram. RelE concentrations from top to bottom: 100, 50, 25, 12.5 and 0 nM. Dashed lines indicate fitted values used to obtain the rate constants (see the text). (b) Tryptophan fluorescence analysis of the RelB–RelE interaction. Complex formation was monitored by stoichiometric titration of RelE (0.35 μM) with RelB. RelE tryptophans were excited at 295 nm, and the emission signal was monitored from 315 to 400 nm. The maxima values at approximately 330 nm are represented as fractional change. Each point represents the average of four measurements. Error bars represents the standard deviation.

RelE increased both the affinity (> 10-fold) and the cooperativity of RelB dimers for *relO*.^33,34^ Using quantitative immunoblotting, we estimated that there are 550 to 1100 RelB and 50 to 100 RelE molecules in rapidly growing cells of MG1655 carrying WT *relBE* at its native chromosomal locus (Fig. S3). These measurements were difficult to accomplish due to the low cellular amounts of the proteins, and, to our knowledge, this is the first estimate of the levels of a toxin–antitoxin pair expressed in its native context. RelB was in ≈ 10-fold excess of RelE. This relatively modest excess of RelB can keep RelE inactivated only if RelB binds very tightly to RelE. To address this question quantitatively, we measured the affinity of the RelB–RelE interaction by surface plasmon resonance (SPR) analysis. To this end, we immobilized RelB-Cys on an SPR sensor chip (the extra cysteine in RelB did not change the interaction with RelE; see Materials and Methods). To determine the kinetics of the interaction, RelE was injected over the chip surface containing immobilized RelB-Cys. The result of repeated cycles of association, dissociation and regeneration using increasing concentrations is shown (Fig. 5a). The binding response data were fitted to an equation (Langmuir, Materials and Methods) to obtain estimates for the rate constants *k*_on_ (5.01 × 10^5^ M^− 1^ s^− 1^) and *k*_off_ (1.66 × 10^− ^^4^ s^− 1^). The apparent dissociation constant *K*_d_ (*k*_off_*/k*_on_) of 0.33 nM was similar to the strong binding affinities reported for other TA systems.^42,43^ As described in the Supplemental Material, these data allowed us to estimate that, on average, there is less than one free RelE molecule per cell in rapidly growing cells.

Recently, we reported that RelB and RelE form a RelB_2_·RelE complex,^33^ whereas data obtained elsewhere favored a RelB_2_·RelE_2_ complex.^34^ To obtain more information on the stoichiometry of the RelB·RelE complex in solution, we measured tryptophan fluorescence of RelE (RelB does not contain tryptophan) upon titration with RelB (Fig. 5b). As shown, the increase in fluorescence upon complex formation leveled off at a RelB/RelE ratio of ∼ 2. This result supports the formation of a (RelB_2_·RelE)_*n*_ complex. To obtain more independent estimates of the stoichiometry, we also performed amino acid analysis of purified RelB·RelE complex (Table S2), which was preformed *in vivo* (Supplemental Material). The experimentally determined number of residues were clearly in favor of a 2:1 ratio of RelB/RelE.

### RelB displays functional interaction with Lon

**Fig. 6:**
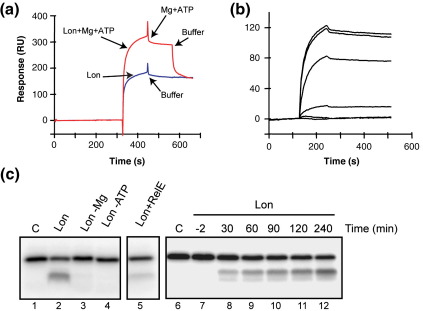
RelB is degraded by Lon protease. (a and b) SPR analysis. RelB-Cys was immobilized on a CM5 sensor chip and Lon was injected at a concentration of 200 nM with (red sensorgram) or without (blue sensorgram) MgCl_2_ (8 mM) and ATP (2 mM) in the buffer as indicated by arrows in the figure. At time ∼ 450 s, the dissociation phase was initiated with (red sensorgram) or without (blue line) MgCl_2_ and ATP in the buffer. Finally, a second dissociation phase at time ∼ 550 s was initiated by changing to buffer without MgCl_2_ and ATP (red sensorgram only). For kinetic measurements, the following Lon concentrations were used: from top to bottom, 100, 50 nM, 25 nM, 12.5 and 0 nM. (c) *In vitro* degradation of HMK-RelB. Lon and ^32^P[HMK-RelB] were incubated at 37 °C for 120 min (lanes 1–5) or the times indicated (lanes 6–12) in reaction buffer with or without the components specified and subjected to SDS-PAGE and phosphoimaging. In control reactions (lanes 1 and 6) Lon was omitted.

The ATP-dependent protease Lon degrades RelB *in vivo*.^2^ To analyze the RelB–Lon interaction, we performed SPR analysis of the purified proteins. Injection of Lon-His_6_ (Lon) over a chip surface containing immobilized RelB-Cys resulted in a binding response with a slow dissociation phase (Fig. 6a). This interaction was further stimulated by including the Lon cofactors, ATP and Mg^2+^, in the buffer. Interestingly, removal of ATP and Mg^2+^ resulted in fast dissociation of additional bound Lon back to the level obtained without the addition of cofactors. A weak stimulatory effect of Mg^2+^ alone was detected (data not shown). This indicates that most of the effect observed when including ATP and Mg^2+^ in the buffer can be ascribed to ATP.

To determine the kinetic constants of the interaction, increasing concentrations of Lon were injected over the sensor chip surface containing immobilized RelB-Cys (Fig. 6b). The rate constant *k*_off_ was quite low (2.34 × 10^− ^^4^ s^− 1^) but we failed to obtain a reliable fit for the association rate constant *k*_on_. Nevertheless, we estimate the apparent dissociation constant *K*_d_ to be less than 2 nM.

Finally, we investigated the functionality of the interaction between Lon and RelB using an *in vitro* degradation assay. Here we used a RelB variant containing an N-terminal tag for heart myosin kinase (HMK-RelB) to allow ^32^P labeling of the protein for detection of full-length RelB as well as possible degradation products in a quantitative manner. Incubation of Lon with HMK-RelB resulted in degradation of HMK-RelB in an ATP- and Mg^2+^-dependent manner (Fig. 6c, lanes 1–4). Prior formation of a complex between HMK-RelB and RelE reduced the degradation rate (Fig. 6c, lane 5). The *in vitro* half-life of HMK-RelB was long (> 60 min) (Fig. 6c, lanes 7–12). Since the degradation rate slowed significantly after 60 min, this could reflect either depletion of ATP or inactivation of Lon. We did not obtain any effects of including an ATP regeneration system or by using native RelB (data not shown).

## Discussion

RelB is the crucial regulator of the model *relBE* locus. Therefore, we investigated in detail the molecular interactions of the RelB antitoxin with respect to (i) the *relO* operator DNA, (ii) interaction with RelE and (iii) interaction with the ATP-dependent protease Lon.

To delineate the RelB DNA binding and/or co-repressor interaction space, we used mutagenic PCR to obtain *relB* mutants that, when cloned in our *lacZ* reporter system (Fig. 1), conferred derepression of the *relBE* promoter. The amino acid changes in RelB mapped to distinct and important domains within the N-terminal DNA-binding RHH motif.^34,44^ At the β-stranded N-terminus forming an antiparallel β-sheet with a second RelB monomer (Fig. 1c and d), we obtained a mutation of isoleucine I8 into a threonine. Together with the conserved hydrophobic amino acids I4 and L6, this residue constitutes an important element of the hydrophobic core of the RHH domain. In chemical cross-linking assays, the RelB^I8A^ mutant formed dimeric and tetrameric species, similar to WT RelB (Fig. S1). By inference, I8 probably plays a more direct role in DNA interaction, since its multimerization properties appeared similar to those of WT RelB. Consistently, NMR titration studies of RelB revealed major chemical shift perturbations of I8 upon binding to operator DNA.^34^

Unexpectedly, the β-strand residues N5 and R7 that are engaged in nucleotide base specific contacts^34^ were not obtained in the screen. We therefore constructed the RelB^R7A^ variant and thereby confirmed the importance of this basic residue in our *in vitro* and *in vivo* operator-binding assays (Fig. 2).

The DNA binding surface of RelB displays an overall positive electrostatic potential, which is primarily maintained by residues R7 and K13. Several mutants obtained in our screen resulted in the RelB^K13E^ variant, indicating the importance of the charge contribution from this residue. According to the RelB–DNA model, K13 side chains are in close proximity to the DNA phosphate backbone on either side of DNA (Fig. 1d), implying potential electrostatic backbone contacts. In addition, K13 displays major chemical shift perturbations upon binding to operator DNA.^34^

In RHH proteins, β-sheet binding to DNA major groove by nucleotide base specific contacts are assisted by further nonspecific contacts to the DNA phosphate backbone.^44^ These contacts are made specifically by protein-backbone amide nitrogen atoms at the N-terminus of helix α2. We also obtained mutations in S28 of RelB (S28F/H) in our screen and S28L/R changes introduced by site-directed mutagenesis affected operator binding and *relBE* autoregulation, as expected (Fig. 2). In the crystal structure of CopG bound to DNA, hydrogen bonds between side chains of S29 at the N-terminus of helix α2 and phosphate groups of DNA further sustain this backbone interaction^45^ and thus provide a possible explanation for the conservation of serine or threonine residues at this position. Finally, the S28L/R RelB mutants formed dimeric and tetrameric species in chemical cross-linking assays (Fig. S1). This indicates that unlike similar amino acid changes in the dimer–dimer interface of Arc repressor (S35L/R) defective in cooperative operator binding, RelB tetramerization includes additional contacts, which potentially could involve the flexible C-terminus.^34,41^

In the loop region between helices α1 and α2, the conserved G-X-S/T/N motif is critical for the correct positioning of the N-terminus of helix α2 to make anchoring contacts to the DNA phosphate backbone.^44^ Consistently, a V25G mutation led to promoter derepression (Fig. 1b) and a site-directed G24P mutation resulted in a hyperlabile RelB mutant defective in autoregulation and operator binding (data not shown). Taken together, our genetic approach was very useful in the construction of RelB mutants, since we picked up amino acid changes in most of the signature residues within the RHH DNA-binding domain.

Recently, we showed that RelB in complex with RelE binds cooperatively to separate half-sites in *relO*, suggesting that two RelB_2_·RelE heterotrimers occupy both sites simultaneously.^33^ Interaction occurred exclusively via RelB, as RelE alone or in complex with RelB did not alter the DNase I protection pattern. Here we performed a mutational analysis of the *relO* operator to determine nucleotide bases within the operator half-sites critical for specific binding of RelB dimers. We found, with a resolution of two nucleotides, that the central four nucleotides within the hexad core sequence [A/T]TGT[A/C]A are highly critical for autorepression (Fig. 3). In addition, we found that no spacing could be accommodated between the two 12-bp pseudo palindromic and perfect palindromic half-sites, which is in accordance with a cooperative binding mode. Consistently, RelB and the RelB_2_·RelE complex confer hydroxyl radical backbone protection at TG and TTG sequences in the left and right half-sites, respectively (Figs 3 and 4).

Previous studies showed that free RelE inhibits protein synthesis by inducing cleavage of mRNAs positioned in the ribosomal A-site.^5^ This activity of RelE is counteracted by direct protein–protein interaction to antitoxin RelB.^5,31^ The interaction between RelB and RelE depends on the flexible and proteolytically cleavable C-terminus of RelB.^46^ We obtained estimates of the kinetic rate constants of the RelB–RelE interaction (Fig. 5), which confirmed that RelB and RelE form a highly stable complex with an *in vitro* half-life of approximately 70 min. Based on our estimates of the total cellular amounts of RelB and RelE (Fig. S3) and our SPR interaction data, we estimated that the cellular level of free RelE is probably lower than one molecule per cell. This low level must be due to both the negative transcriptional control loop and the tight complex formation between RelB and RelE. Since overexpression of RelE very efficiently inhibits translation,^30,31^ the level of free RelE must be very low in rapidly growing cells and our estimate is thus consistent with this inference.

In agreement with previously published results,^33^ our data obtained here favor a stoichiometry of 2:1 for the RelB–RelE complex (Fig. 5b and Table S2). In two separate studies, analytical gel-filtration analysis determined the mass of the RelB·RelE complex to be 47 and 49 kDa, respectively.^34,46^ This is close to the calculated mass of a (RelB·RelE)_2_ heterotetramer. Indeed, a heterotetramer was obtained in the crystal structure of an archaeal RelB·RelE complex.^18^ In support of a 2:1 stoichiometry, we have analytical gel-filtration data that show that free RelE is a monomer (data not shown), and from chemical cross-linking we have evidence that RelB forms dimers and tetramers in solution (Fig. S1). This is in agreement with additional analysis using differential scanning calorimetry (Fig. S4). Thus, our data favor a RelB_2_·RelE complex as the major species in solution. However, TA stoichiometries are highly dynamic and interconversion between heterotrimer and heterotetramer species allows for increased capacity of the antitoxin to neutralize its cognate toxin and also provides a mechanism for TA ratio-dependent derepression of their cognate promoters.^33,47,48^ We suggested previously that RelB_2_·RelE is the primary repressor complex that binds at *relO* and that excess RelE leads to the formation of a RelB_2_·RelE_2_ complex that does not bind to *relO*, thereby explaining mechanistically how excess of RelE derepresses *relBE* transcription.^33^

Activation of RelE is achieved through ATP-dependent RelB degradation by the Lon protease.^2,23^ Here we provide direct evidence for a strong interaction between Lon and RelB *in vitro* (Fig. 6a). Moreover, we found that the interaction was enhanced by ATP and to a lesser degree by magnesium. The nature of this interaction is not known; however, overexpression of a proteolytically inactive Lon mutant sequesters efficiently Lon substrates and thereby complements the mutant phenotypes of a *lon* mutant.^49^ Thus, initial substrate binding is important for Lon function and is in good agreement with the stable complexes we obtained using SPR analysis. We also confirmed that Lon degrades RelB *in vitro* in a magnesium- and ATP-dependent manner (Fig. 6b). This degradation is likely to be initiated at the proteolytically unstable C-terminus of RelB that is protected by RelE.^46^ Prior formation of RelB_2_·RelE complexes by addition of a molar excess of RelE reduced degradation, consistent with the proposal that RelE interacts with the C-terminus of RelB and thereby protects RelB from degradation by Lon.

**Fig. 7:**
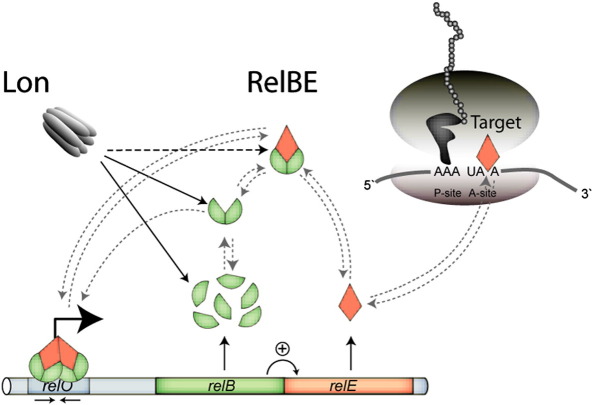
Model explaining regulation of the *relBE* locus. The *relBE* operon has a typical TA organization. The first gene encodes the RelB antitoxin (green) and the second gene encodes mRNA interferase and co-repressor RelE (red). RelB is produced in ∼ 10 fold excess of RelE due to coupled translation (bent arrow, +). RelB monomers readily form dimers via their N-terminal RHH domain and interact with RelE monomers to form a tight nontoxic complex. During exponential growth, RelE is exclusively bound by RelB. This complex forms a heterohexamer when bound to *relO*, which involves cooperativity mediated by co-repressor RelE. The repression complex bound to its operator overlapping the − 10 box in the promoter region prevents transcription initiation of RNA polymerase and results in a low level of *relBE* expression. In steady-state and upon stress induced growth arrest, the ATP-dependent protease Lon degrades RelB at a high rate. Lon degrades RelB in its free states and in complex with RelE. A reduction in the global rate of translation as a consequence of stress shifts the RelE equilibrium toward free RelE as the level of RelB declines. This shift in the ratio between RelB and RelE results in derepression of the promoter due to loss of cooperativity in the repression complex. Increased transcription may contribute to a decrease in the RelB/RelE ratio in concert with enhanced activity of Lon. As a result RelE is free to act on its target, the ribosomal A-site, to promote codon-specific cleavage of mRNA. This in turn reduces energy consumption in the cell and may yield a lower level of translation errors.

In this study, we have characterized multiple important molecular interactions within the RelBE system in *E. coli*: interaction of RelB with RelE, preventing its mRNA interferase activity and essential for efficient autoregulation; interaction of RelB with Lon, regulating its metabolic turnover and activation of RelE; interaction of RelB with *relO* DNA, required for autoregulation of the *relBE* operon. Combined, these interactions keep the system in the OFF-state during steady-state growth along with a derepressor mechanism and translational coupling ensuring RelB is kept in excess to RelE (Christensen and Gerdes, unpublished data). Upon nutritional stress, such as amino acid starvation, *relBE* is activated and reduces the global rate of translation.^2^ Shortly after onset of starvation, the RelB/RelE ratio is reduced. This change leads to release of the RelB_2_–RelE complex and derepression of the strong *relBE* promoter. Unfortunately, we do not have direct evidence for the notion that this trigger mechanism is important for the biological function of *relBE*. However, the trigger mechanism is conserved in nonhomologous TA loci,^15,47,50,51,52^ arguing that indeed it is important. One function may be to promote rapid induction of *relBE* transcription at the onset of nutritional stress such as amino acid starvation. The regulatory interactions with the RelBE system are summarized in the model shown in Fig. 7 and further explained in the figure legend.

The biological function of chromosomal TA loci is currently a matter of debate. However, accumulating evidence suggests that at least some of them function to adjust the global rates of translation and replication to a given supply of nutrients from the environment.^1^ Accordingly, in support of the stress response hypothesis, transcription of a number of TA loci was induced by heat shock in *Sulfolobus sulfataricus*^53^ and by exposure to chloroform in *Nitrosomonas europaea*.^54^

## Materials and Methods

### Media, antibiotics, strains and plasmids

Luria–Bertani (LB) and 2 × YT medium were prepared as described.^55^ When required, LB broth or LB plates were supplemented with ampicillin (30 or 100 μg/ml; Amp). X-gal was added to indicator plates to a final concentration of 40 μg/ml. Expression of proteins from the pA1/O4/O3 or pBAD promoters was induced by 1 mM isopropyl-β-d-thiogalactopyranoside (IPTG) or 0.2% arabinose, respectively. Strains and plasmids are listed in Table 1. The Top10 strain was used as the standard host strain for cloning and the resulting plasmids were subsequently transformed into relevant strain backgrounds.

### Mutagenic PCR and cassette mutagenesis

To synthesize DNA fragments carrying random mutations between codon 4 and codon 75 of *relB*, 0.1 mM MnCl_2_ was added to an otherwise standard Taq PCR reaction containing primers relB-mut-BamHI-f and relB-mut-XbaI-r using pMO2541 as template. The resulting PCR fragment (∼ 0.23 kb) was inserted into BamHI–XbaI-digested pMO2541 vector and ligated. pMO2541 and its mutant derivatives were plated onto LB agar + Amp (30 μg/ml) + X-gal (40 μg/ml) and screened for dark blue (derepressed) colonies among a background of pale colonies (repressed).

### Protein expression and purification

The RelB His_6_–RelE complex formed *in vivo*, RelB (WT and mutant alleles) and His_6_-RelE proteins were purified using the strains and plasmids indicated in Table 1 and according to the protocol described previously.^58^ Lon-His_6_ was purified from strain LVM781/pBAD24-lon-his_6_. Briefly, a 5-ml overnight culture was diluted into 1 L of 2 × YT medium containing ampicillin (100 mg/ml; Amp) and was cultured at 30 °C. At OD_450_ ∼ 0.5, the expression was induced by arabinose for 3 h. The culture was harvested by centrifugation and resuspended in ice-cold buffer A (50 mM NaH_2_PO_4_, 0.3 M NaCl, 10 mM imidazole, 5 mM β-mercaptoethanol, pH 8). The cells were disrupted by passing them three times through a French press cell and the soluble fraction was isolated by centrifugation at 48,400*g* for 30 min. The cleared lysate was incubated with Ni–NTA agarose resin (Qiagen) in batches according to manufacturer instructions and subsequently loaded onto a gravity flow column at 4 °C. The Ni–agarose resin was washed extensively in buffer B (50 mM NaH_2_PO_4_, 1 M NaCl, 60 mM imidazole, 1 mM β-mercaptoethanol, pH 8). Finally, Lon-His_6_ was eluted in buffer B and 250 mM imidazole and diluted threefold before dialysis storage buffer [50 mM Tris (pH 7.5), 0.2 M KCl, 1 mM ethylenediaminetetraacetic acid (EDTA), 10% glycerol, 1 mM DTT].

Each purified protein was at least 95% pure as estimated by silver staining of SDS-PAGE gels. The molecular masses of all proteins used in this study were verified by matrix-assisted laser-desorption/ionization time-of-flight mass spectrometry.

### β-Galactosidase assay

Activity of the various *relBE-lacZ* derivatives listed in Table 1 was measured according to Miller^59^ and as described previously.^33^

### Electrophoretic mobility shift assay and hydroxyl radical footprinting

Gel-shift assays were carried out using the protocol described previously.^33^

Hydroxyl radical footprinting was carried out essentially according to Tullius *et al.*^60^ Primer labeling (^32^P), PCR and binding reactions were prepared as described.^33^ Each binding reaction was made in a total volume of 35 μl. To initiate generation of hydroxyl radicals and, thus, DNA backbone cleavage, 5 μl of a freshly prepared mixture of 0.1 mM (NH_4_)_2_Fe(SO_4_)_2_·6H_2_O and 0.2 mM EDTA was deposited on the inner wall of an Eppendorf tube along with 5 μl of 0.6% H_2_O_2_ and 5 μl of sodium ascorbate (10 mM). The cutting reagents were mixed with the protein–DNA solution and incubated at 37 °C for 1 min. The reaction was stopped by the addition of 21 μl of stop solution (23.8 mM thiourea, 152 mM EDTA, 0.2 mg/ml salmon sperm DNA, 2% glycerol). The resulting fragmented DNA was extracted once in phenol and once in chloroform, precipitated and washed in ethanol and separated on 6% denaturing acrylamide gels along with a G+A sequencing ladder (see below). The gel was dried and finally subjected to phosphoimaging using a Typhoon Trio scanner (GE Healthcare).

### Maxam–Gilbert G+A sequencing

Radiolabeled PCR (100 nM) was chilled on ice for 5 min with 4 μg of sheared salmon sperm (ss) DNA in a total volume of 24 μl. To the sample was then added 4 μl of piperidine formate (1 M, pH 2) and incubated at 37 °C for 15 min. The reaction was terminated by the adding 240 μl of stop reagent (0.3 M sodium acetate, pH 7) and the DNA was precipitated by adding 750 μl of 96% ethanol, chilled at − 80 °C for 10 min and collected by centrifugation. The latter step was repeated twice, once with 0.1 M sodium acetate (pH 4.5) and once in 96% ethanol. Then 100 μl of piperidine (1 M) was added to the DNA pellet and incubated at 90 °C for 30 min. The DNA solution was snap-frozen in liquid nitrogen and dried in a speed-vac overnight. Finally, the pellet was resuspended in 10 μl of water and dried in a speed-vac twice and resuspended in 80% formamide loading buffer. Sequencing reactions were separated alongside the corresponding hydroxyl radical reactions as described above.

### Surface plasmon resonance analysis

SPR analyses of RelB–RelE and RelB–Lon interactions were carried out on a Biacore 3000 instrument (Biacore AB) equipped with a CM5 sensor chip. In short, Approximately 40 RU RelB-Cys was immobilized with ligand thiol coupling [4 min NHS/EDC, 4 min PDEA, short pulses of RelB-Cys in 10 mM NaAc (pH 3.5), reduced and desalted on Poros R1, 4 min cysteine]. His_6_-RelE or Lon-His_6_ at concentrations (monomeric) indicated in the figure was injected over immobilized RelB-Cys in series with a nonderivatized reference control flow cell at a flow rate of 60 μl/min. After each injection, RelE/Lon was released from the surface by two consecutive 30-s injections of 3 M guanidine HCl in 50% running buffer (HBS-EP: 10 mM Hepes, 150 mM NaCl, 0.005% Tween 20, 3 mM EDTA, pH 7.4). The response of the reference flow cell was subtracted from the response of the RelB-Cys flow cell and then from the response of a buffer injection. In order to obtain the rate constants, double reference subtracted sensorgrams were fitted (least sum of squared residuals) to a model of 1:1 binding (Langmuir binding) using the BIAevaluation 3.1 software package. Calculations of stoichiometry were carried out as described.^33^

### Tryptophan fluorescence measurements

Fluorescence experiments were carried out using an LS 55 fluorescence spectrometer (Perkin Elmer) at 25 °C. Tryptophan fluorescence of His_6_-RelE protein (0.35 μM) was measured before and after addition of increasing molar amounts of RelB. All titrations were performed in phosphate-buffered saline (pH 7.4). Complexes were allowed to form by gently mixing the components for 5 min in the cuvette with the aid of a small stir bar. Emission spectra were taken from 315 to 400 nm with excitation at 295 nm using a slit width of 10 nm. Data were collected by integration of five consecutive scans at a scan speed of 900 nm/min. Each data point represents the peak value (at approximately 330 nm) for the mean of five scans expressed as fractional change.

### Lon/RelB degradation assay

To monitor the degradation of RelB, the ATP-dependent protease Lon (0.6 μM) was added to a reaction buffer [50 mM Tris–HCl (pH 8), 4 mM ATP, 7.5 mM MgCl_2_]. Prior to the experiments, HMK-RelB was labeled with ^32^P on the N-terminal HMK-tag (RRASV) using the PKAce kit (Novagen) according to the manufacturer's instructions. Formation of complexes between HMK-RelB and His_6_-RelE was achieved by preincubating 0.13 μM HMK-RelB with 0.52 μM His_6_-RelE at ambient temperature for 20 min. HMK-RelB (0.13 μM), free or in complex, was finally added to the reaction mixture in a final volume of 20 μl and incubated at 37 °C for the times indicated in the figure. Degradation was stopped by adding SDS-loading buffer and boiling of the samples for 5 min at 95 °C. The proteins were separated by SDS-PAGE using 16% Tris–tricine gels^61^ and visualized by phosphoimaging of the dried gels.

### Modeling of the RelB–DNA complex

Modelling of RelB–DNA was done using structures of the N-terminal domain of RelB (RelB_1–50_) [Protein Data Bank (PDB) code 2k29] and of the Arc–DNA complex (PDB 1bdt) as template. The backbone of the RelB_1–50_ dimer was superimposed onto the template RHH dimer structure by using the VMD plugin multiseq.

## Figures and Tables

**Table 1:** Bacterial strains and plasmids used in this study

Strains/plasmids	Genotype	Source/reference
*Strains*		
MG1655	Wild-type *E. coli*	56
MG1	MC1000Δ*relBEF::kan*	2
SC34	MG1655Δ*relBEF*	36
SG22095	Δ*lac rcsA166*Δ*aphA lon146*::*tet*	Susan Gottesman
LVM781	F^−^*mcrA*Δ(*mrr-hsd*RMS-*mcrBC*) φ80*lac*ZΔM15 Δ*lac*X74 *deo*R *rec*A1 *ara*D139 Δ(*ara-leu*)7697 *gal*U *gal*K *rps*L (Str^R^) *end*A1 *nup*G λ^−^	L. Van Melderen
Top10	BL21(DE3) mutant strain	Invitrogen
C41 (DE3)	Wild-type *E. coli*	57

*Plasmids*		
pSC2524	pMG25, PA1/O4/O3::*SD*_*opt*_::*relB*:: *SD*_*opt*_::*his6*::*relE*	Laboratory collection
pMO207	pMG25, PA1/O4/O3::*SD*_*opt*_::*hmk-relB*:: *SD*_*opt*_::*his6*::*relE*	This work
pMO213	pMG25, PA1/O4/O3::*SD*_*opt*_::*relB-cys*:: *SD*_*opt*_::*his6*::*relE*	This work
pOU253	Mini-R1, *bla*, *lacZYA*, translational fusion vector	Laboratory collection
pKG4001	pOU254, *bla, relB 5′*::*lacZYA*	19
pMO2534	pOU253, *bla, relBE*::*lacZYA*	This work
pMO2535	pOU253, *bla, relBE*^*R81A*^::*lacZYA*	This work
pMO2540	pOU253, *bla, BamHI-relB-XbaI-relE-KpnI*::*lacZYA*	This work
pMO2541	pOU253, *bla, BamHI-relB-XbaI-relER81A-KpnI*::*lacZYA*	This work
pMGJ4004	pOU254, *bla, relO(WT), relBE*^*R81A*^::*lacZYA*	This work
pMO259	pOU254, *bla, relO1, relBE*^*R81A*^::*lacZYA*	This work
pMO277	pOU254, *bla, relO1, relB 5′*::*lacZYA*	This work
pMO260	pOU254, *bla, relO2, relBE*^*R81A*^::*lacZYA*	This work
pMO278	pOU254, *bla, relO2, relB 5′*::*lacZYA*	This work
pMO261	pOU254, *bla, relO3, relBE*^*R81A*^::*lacZYA*	This work
pMO279	pOU254, *bla, relO3, relB 5′*::*lacZYA*	This work
pMO262	pOU254, *bla, relO4, relBE*^*R81A*^::*lacZYA*	This work
pMO280	pOU254, *bla, relO4, relB 5′*::*lacZYA*	This work
pMO263	pOU254, *bla, relO5, relBE*^*R81A*^::*lacZYA*	This work
pMO281	pOU254, *bla, relO5, relB 5′*::*lacZYA*	This work
pMO264	pOU254, *bla, relO6, relBE*^*R81A*^::*lacZYA*	This work
pMO282	pOU254, *bla, relO6, relB 5′*::*lacZYA*	This work
pMO265	pOU254, *bla, relO7, relBE*^*R81A*^::*lacZYA*	This work
pMO283	pOU254, *bla, relO7, relB 5′*::*lacZYA*	This work
pMO266	pOU254, *bla, relO8, relBE*^*R81A*^::*lacZYA*	This work
pMO284	pOU254, *bla, relO8, relB 5′*::*lacZYA*	This work
pMO267	pOU254, *bla, relO9, relBE*^*R81A*^::*lacZYA*	This work
pMO285	pOU254, *bla, relO9, relB 5′*::*lacZYA*	This work
pMO268	pOU254, *bla, relO10, relBE*^*R81A*^::*lacZYA*	This work
pMO286	pOU254, *bla, relO10, relB 5′*::*lacZYA*	This work
pMO269	pOU254, *bla, relO11, relBE*^*R81A*^::*lacZYA*	This work
pMO287	pOU254, *bla, relO11, relB 5′*::*lacZYA*	This work
pMO270	pOU254, *bla, relO12, relBE*^*R81A*^::*lacZYA*	This work
pMO288	pOU254, *bla, relO12, relB 5′*::*lacZYA*	This work
pMO271	pOU254, *bla, relO13, relBE*^*R81A*^::*lacZYA*	This work
pMO289	pOU254, *bla, relO13, relB 5′*::*lacZYA*	This work
pMO272	pOU254, *bla, relO14, relBE*^*R81A*^::*lacZYA*	This work
pMO290	pOU254, *bla, relO14, relB 5′*::*lacZYA*	This work
pMO273	pOU254, *bla, relO15, relBE*^*R81A*^::*lacZYA*	This work
pMO291	pOU254, *bla, relO15, relB 5′*::*lacZYA*	This work
pMO274	pOU254, *bla, relO16, relBE*^*R81A*^::*lacZYA*	This work
pMO292	pOU254, *bla, relO16, relB 5′*::*lacZYA*	This work
pMO237	pMG25, PA1/O4/O3::*SD*_*opt*_::*relB*^*R7A*^:: *SD*_*opt*_::*his6*::*relE*	This work
pMO238	pMG25, PA1/O4/O3::*SD*_*opt*_::*relB*^*I8A*^:: *SD*_*opt*_::*his6*::*relE*	This work
pMO239	pMG25, PA1/O4/O3::*SD*_*opt*_::*relB*^*K13A*^:: *SD*_*opt*_::*his6*::*relE*	This work
pMO240	pMG25, PA1/O4/O3::*SD*_*opt*_::*relB*^*S28L*^:: *SD*_*opt*_::*his6*::*relE*	This work
pMO241	pMG25, PA1/O4/O3::*SD*_*opt*_::*relB*^*S28R*^:: *SD*_*opt*_::*his6*::*relE*	This work
pMO244	pOU254, *bla, relB^R7A^relE^R81A^*::*lacZYA*	This work
pMO245	pOU254, *bla, relB^I8A^relE^R81A^*::*lacZYA*	This work
pMO246	pOU254, *bla, relB^K13A^relE^R81A^*::*lacZYA*	This work
pMO249	pOU254, *bla, relB^S28L^relE^R81A^*::*lacZYA*	This work
