# Supplementary material for: RelB and RelE of Escherichia coli Form a Tight Complex That Represses Transcription via the Ribbon–Helix–Helix Motif in RelB
Source: J Mol Biol. 2009 Nov 27;394(2):183–96. doi: 10.1016/j.jmb.2009.09.006 (PMC2812701; doi:10.1016/j.jmb.2009.09.006)

**Supplemental Material to Overgaard *et al.***

*Construction of plasmids containing relO operator mutations*

Sixteen mini-R1 derivatives (pMO259-274) were constructed, each containing different mutated *relO* fragments cloned in front of *relBE^R81A^*-*lacZ* in the transcriptional fusion vector pMGJ4004 (see Table 1). Operator dinucleotide transversions were generated by three rounds of PCR and introduced into pMGJ4004. PCR 1: primers relB-80f-EcoRI and relB-O1r through relB-O16r (see Table 2) on pMGJ4004; PCR 2: primers relB-O1f through O16f and relB2-XhoI; PCR 3: primers relB-80f-EcoRI and relB2-XhoI on PCR 1+2. The resulting 0.23 kb fragments (PCR 3) were each inserted into pMGJ4004 using the restriction sites *EcoR*I and *Xho*I. To correct for altered promoter strength caused by the operator mutations introduced, drop-out derivatives were made for each construct lacking *relB 3`-relE^R81A^* carried on a *Xho*I-*Bam*HI fragment. All plasmids were subsequently transformed into the *E. coli* strain MG1.

*Construction of HMK-RelB and RelB-Cys-RelE expression plasmids*

pMO207/pMO213: *hmk-relB* (encodes an N-terminal RRASV-tag on RelB) was amplified from pBD2430 using primers relB1-2524-ntd-hmk and relB2-2524 and relB-cys (encodes a RelB variant with a C-terminal cysteine residue) was amplified from pBD2430 using primers relB1-2524 and relB2-2524-ctd-cys. PCR products were digested with *Eco*RI and *Bam*HI and ligated to similar digested pSC2524 expression vector DNA. The resulting mutant derivatives of pSC2524 were transformed into *E. coli* Top10.

*Construction of expression plasmids for mutant alleles of the RelB RHH motif*

The plasmids, pMO237, pMO238 and pMO239 (Table 1) were constructed in one step by amplifying mutant relB using either of the forward primers relB1-2524-R7A, relB1-2524-I8A and relB1-2524-K13A together with the reverse primer relB2-2524 and pSC2524 as template. The resulting PCR products were cut with *Eco*RI and *Bam*HI and re-inserted into the pSC2524 vector at the *EcoR*I and *BamH*I sites. The plasmids, pMO240 and pMO241 were generated by three rounds of PCR and introduced into pSC2524. PCR 1: primers relB1-2524 and the primer relB2-S28L or relB2-S28R (see Table S1) on pBD2430; PCR 2: primer relB2-2524 and the primer relB1-S28L or relB1-S28R; PCR 3: primers relB1-2524 and relB2-2524 on PCR 1+2. The resulting fragments (PCR 3) were each inserted into pSC2524 using the restriction sites *Eco*RI and *Bam*HI. The plasmids, which are thus mutant derivatives of pSC2524, were finally transformed into the *E. coli* strains Top10 or C41 (DE3).

*Construction of relBE^R81A^-lacZ fusion derivatives with mutant alleles of the RelB RHH motif*

To measure the DNA-binding activity of the RelB mutants *in vivo* the mutant alleles of pMO237-242 were moved into pMGJ4004 as follows: PCR 1: primers relB1 and relB2-Xho on pMO237-242 containing each of the six mutations; PCR 2: primer pOU254-CW and relB2 on pMGJ4004; PCR 3: primers pOU254-CW and relB2-Xho on PCR 1+2. The resulting fragments (PCR 3) were each inserted into pMGJ4004 using the restriction sites *Eco*RI and *Xho*I and transformed into strain MG1.

*Construction of plasmids for cassette mutagenesis of relB*

pMO2534: A *relB-relE* fragment was amplified by PCR using the primers relB-XhoI-f and relE2-KpnI and pBD2430 as template. The PCR fragment was digested with *Xho*I and *Kpn*I and inserted into *Xho*I -*Kpn*I digested pKG4001 generating pMO2534. pMO2535: A *relB-relE* fragment was amplified by PCR using the primers relB1-XhoI and relE2-KpnI and pMGJ4004 as template. The PCR fragment was digested with *Xho*I and *Kpn*I and inserted into *Xho*I-*Kpn*I digested pKG4001 generating pMO2535. MO2536: pMO2534 was digested with *Bam*HI and ligated to a linker 253-mut-BamHI-f, 253-mut-BamHI-r that mutates the *Bam*HI restriction site in frame between *relE* and *lacZ* while introducing a new EagI site. MO2537: pMO2535 was digested with *Xho*I and *Kpn*I and the resulting 463 bp *relB-relE* fragment was inserted into *Xho*I -*Kpn*I digested pMO2536 generating pMO2537. pMO2538 and pMO2539: A silent mutation creating a *Xba*I site was introduced into the 3`end of *relB.* PCR 1: primers relB -80-EcoRI and relB-mut-XbaI-r on pMO2536 (PCR 1); PCR 2: primers relB-mut-XbaI-f and relE2-KpnI; PCR 3: primers relB -80-EcoRI and relE2-KpnI. The resulting 0.65 kb fragment (PCR 3) was inserted into pMO2536 and pMO2537 using the restriction sites *Eco*RI and *Kpn*I thus resulting in pMO2538 and pMO2539. pMO2540 and pMO2541: A silent mutation that creates a *Bam*HI site was introduced into the 5`end of *relB.* PCR 1: primers relB -80-EcoRI and relB-mut-BamHI-r on pMO2536 (PCR 1); PCR 2: primers relB-mut-BamHI-f and relE2-KpnI; PCR 3: primers relB -80-EcoRI and relE2-KpnI on PCR 1+2. The resulting 0.65 kb fragment (PCR 3) was inserted into pMO2538 and pMO2539 using the restriction sites *Eco*RI and *Kpn*I thus resulting in pMO2540 and pMO2541. The plasmids were transformed into the *E. coli* strain MG1. All cloned DNA fragments that were synthesized by PCR were subsequently verified by DNA sequencing.

*Amino acid analysis*

The RelB-RelE complex was purified as described ^1^ and further purified by size-exclusion chromatography performed at room temperature in PBS buffer pH 7.4 on a Superdex 75 prep grade 16/90 size-exclusion column operated by an ÄKTA FPLC (Amersham Biosciences, Inc., Uppsala, Sweden. The complex eluted as a single peak that was collected and a total of 2 μg of purified RelB-RelE complex was finally subjected to triplicate determinations of amino acid composition according to^2^.

*Chemical cross-linking of RelB*

Bis sulfosuccinimidyl suberate (BS^3^) (Pierce) was added to reactions at a final concentration of 0.5 and 5 mM. RelB proteins were diluted to 35 µM in PBS buffer in a final reaction volume of 15 μl. Reactions were incubated at room temperature for 1 h and stopped by the addition of 1 μl of 1 M Tris-HCl (pH 7.5) followed by 5.5 μl 4 × LDS loadingbuffer (Invitrogen) and 1 μl 1 M DTT. The samples were heated at 95ºC for 2 min and analyzed by SDS-PAGE using 4-12% Novex Bis-Tris precast gels (Invitrogen) and MES SDS running buffer (50 mM MES, 50 mM Tris-base, 0.1% SDS, 1 mM EDTA) according to manufacturer. Protein bands were visualized by Coomassie Blue gel staining.

*Electrophoretic Mobility Shift Assay (EMSA) and Immunoblot analysis*

EMSA analysis of the *rel* promoter/operator region and immunoblotting of RelB and RelE was carried out as described previously^3^. For quantitative immunoblots, cultures were grown in LB medium at 37°C and 1 ml samples harvested by centrifugation at OD_450_ = 0.4 and stored at -20°C. Equal amounts of input cells were used for SDS-PAGE along with known amounts of purified RelB and His_6_-RelE.

*Differential Scanning Calorimetry (DSC) analysis of RelB*

The thermodynamic properties and the oligomeric state of RelB in solution were studied using DSC. Thermal unfolding of RelB resulted in highly reversible transitions (Fig. S4a, b) independent of the concentration or the scan rate and where more than 95% of the protein contributed to the unfolding endotherm on successive scans. All DSC thermograms between pH 5.5 and 8.5 showed a single unfolding transition. Non-linear fitting of the data to the equilibrium two-state unfolding model (see below) provided the transition temperatures (Tm) and enthalpies (ΔH) for the endotherms (Table S3). The ratio between the model independent calorimetric enthalpy (ΔHcal), calculated from the area under the Cp vs. T curves, and the van’t Hoff enthalpy (ΔH_V_) obtained from fitting the model function to the data, was close to 0.5 in all cases. This is a strong indicative that RelB is a dimer in solution that unfolds directly into the monomeric state without the accumulation of any intermediate. In line with these findings chemical cross-linking also indicated the formation of dimers (Fig. S1). The overall thermodynamic stability of the protein can be described by the free energy of unfolding (ΔG^o^). Fig. S4c shows the temperature dependence of ΔG^o^ for different concentrations of RelB. The fact that RelB is a dimer implies that the equilibrium monomer-dimer in solution would be dependent at all times to the amount of free RelB in living cells. The increase (decrease) in concentration has marked repercussions in the stability of the protein as observed in the changes of the ΔG^o^ vs. T curves (Fig. S4c). RelB behaves as a fully folded protein at 37^o^C in the concentration ranges used in this study (112 μM to 28 μM), however in the cell where the concentration of RelB is $\approx$1-3 µM (Fig. S3), free RelB protein may become partly or completely unfolded.

All calorimetric scans were performed using a MicroCal VP-DSC high-sensitivity differential scanning microcalorimeter with a 0.515 ml cell. Samples were always filtered and degassed for 10 min at 283 K before being examined in the calorimeter. The reversibility of thermally induced transition of RelB (checked by reheating the solution in the calorimeter cell after cooling from the up-scan run) was more than 95% for all measurements. To obtain the thermograms ((C_p_ - C_p_,*_N_*) vs. T curves), the heat capacity of the protein in the initial (folded) state was subtracted from the raw signal corrected for the buffer contribution. All the calorimetric data were analyzed using the MicroCal Origin DSC 7.0 software package.

Analysis of the thermally induced transition:

The analyses of RelB unfolding data suggest the protein unfolds in a two state process (Eq. 1):

where N_n_ represents the RelB dimer (n=2) in the initial folded state, U corresponds to RelB in the final monomeric unfolded state, *K* is the equilibrium constant, C_T_ is the total protein concentration and α is fraction of unfolded protein. According to this model, one can express an average of a physical property, χ, in terms of the corresponding contributions χ_N_ and χ_U_, which characterize native (N) and unfolded (U) states, respectively^4^.

$$\chi=\chi_{N}+\alpha\cdot\left(\chi_{N}-\chi_{D}\right)\text{ }\left(\text{Eq. 2}\right)\text{ }$$

The model function Cp–Cp,N for the DSC signal is the partial molar heat capacity of the protein relative to state N. It can be derived from the first partial derivative of equation (2) on temperature at constant pressure^5,6^.

$$Cp-Cp_{N}=\alpha\Delta Cp+\frac{\left(\Delta H_{C}+\Delta Cp\left(T-T_{1/2}\right)\right)^{2}}{RT^{2}}\left(\frac{1-\alpha}{1-n+\frac{n}{\alpha}}\right)\text{ }\left(\text{Eq. 3}\right)$$

The thermodynamic parameters that characterize the melting curves (ΔH^o^_T1/2_, ΔCp^o^ and T_1/2_) were obtained by fitting the model function for DSC to the experimental temperature profiles using the Levenberg–Marquardt non-linear χ2 regression procedure^7^.

The conformational stability of a protein that unfolds in a two-state fashion, expressed in terms of the corresponding standard free energy change, ΔG_T_^o^, can be obtained by applying the Gibbs–Helmholtz equation:

$$\Delta G^{0}=\Delta G_{1/2}^{0}\frac{T}{T_{1/2}}+\Delta H_{1/2}\left(1-\frac{T}{T_{1/2}}\right)+\Delta C_{P}^{0}\left(T-T_{1/2}-Tln\left(\frac{T}{T_{1/2}}\right)\right)\text{ (Eq. 4)}$$

In this expression T_1/2_ is the melting temperature at which α = 0.5, ΔH^o^_T 1/2_ is the standard enthalpy of denaturation at T_1/2_, and ΔCp^o^ is the difference in heat capacity between the folded and unfolded state assumed to be temperature independent.

References

1. Christensen-Dalsgaard, M., Overgaard, M., Winther, K. S. & Gerdes, K. (2008). RNA decay by messenger RNA interferases. *Methods Enzymol.* **447**, 521-535.

2. Thaysen-Andersen, M., Jorgensen, S. B., Wilhelmsen, E. S., Petersen, J. W. & Hojrup, P. (2007). Investigation of the detoxification mechanism of formaldehyde-treated tetanus toxin. *Vaccine* **25**, 2213-2227.

3. Overgaard, M., Borch, J., Jorgensen, M. G. & Gerdes, K. (2008). Messenger RNA interferase RelE controls relBE transcription by conditional cooperativity. *Mol. Microbiol.* **69**, 841-857.

4. Pace, C. N. (1986). Determination and analysis of urea and guanidine hydrochloride denaturation curves. *Methods Enzymol.* **131**, 266-280.

5. Freire, E. & Biltonen, R. (1978). Estimation of molecular averages and equilibrium fluctuations in lipid bilayer systems from the excess heat capacity function. *Biochim. Biophys. Acta* **514**, 54-68.

6. Lopez, M. M. & Makhatadze, G. I. (2002). Differential scanning calorimetry. *Methods Mol. Biol.* **173**, 113-119.

7. Press, W. H., Teukolsky, S. A., Vetterling, W. T., Flannery, B. P. & Teukolsky, S. A. (1992). *Numerical recipes in C. The art of scientific computing.* *Cambridge: University Press*..
Ref Type: Book, Whole

8. Christensen, S. K., Mikkelsen, M., Pedersen, K. & Gerdes, K. (2001). RelE, a global inhibitor of translation, is activated during nutritional stress. *Proc. Natl. Acad. Sci. U. S. A* **98**, 14328-14333.

| **Table S1.**  DNA oligonucleotides | |
| --- | --- |
| Name | Oligonucleotide sequence (5`-3`) |
| pOU254-CW  pOU254-CCW  RelE2-KpnI  253-mut-BamHI-f  253-mut-BamHI-r  relB1-mut-BamHI  relB2-mut-BamHI  relB1-mut-XbaI  relB2-mut-XbaI  rel -80f  rel +86r  rel +86r-cy5  IR30f  IR30r  rel-80f-EcoRI  relB2-XhoI  relB2-seq  relB2-2524  relB1-2524  relB1-2524-R7A  relB1-2524- I8A  relB1-2524-K13A  relB1-2524-S28L  relB1-2524-S28R  relB1-2524-ntd-hmk  relB2-2524-ctd-cys  relB1-Xho  relB1  relB2  relO-1f  relO-2f  relO-3f  relO-4f  relO-5f  relO-1r  relO-2r  relO-3r  relO-4r  relO-5r  relO -181 f  relO + 192 r  rel-O1f  rel-O1r  rel-O2f  rel-O2r  rel-O3f  rel-O3r  rel-O4f  rel-O4r  rel-O5f  rel-O5r  rel-O6f3  rel-O6r3  rel-O7f  rel-O7r  rel-O8f  rel-O8r  rel-O9f  rel-O9r  rel-O10f  rel-O10r  rel-O11f  rel-O11r  rel-O12f3  rel-O12r3  rel-O13a-f  rel-O13a-r  rel-O14b-f  rel-O14b-r  rel-O15c-f  rel-O15c-r  rel-O16e-f  rel-O16e-r | TAGGGGTTCCGCGCACATTTCCC  TGTGGGATTAACTGCGCGTCGCC  GGGGTACCGAGAATGCGTTTGACCGCCT  GATCTCGGCCGACTAGTA  GATCTACTAGTCGGCCGA  GACATGGGATCCATTAACCTGCGTA  GCAGGTTAATGGATCCCATGTCTTA  CGTGTGACTCTAGATGAACTCTGAT  CAGAGTTCATCTAGAGTCACACGTA  CAGTGATCACCGTTCTTACGACTA  CGCGGCGTAAGAACGCGCTT  XCGCGGCGTAAGAACGCGCTT  TACTTGTAATGACATTTGTAATTACAAGAG  CTCTTGTAATTACAAATGTCATTACAAGTA  CCCCGAATTCCAGTGATCACCGTTCTTACG  CGATATACTCGAGCATGAGA  GCGTCACACGTACTGGCTTAGG  CCCCGGATCCTCAGAGTTCATCCAGCGTCACACGTACTGG  CCCCGAATTCAAAAGGAGGAAAAAACCATGGGTAGCATTAACCTGCGTATTGACG  CCCCGAATTCAAAAGGAGGAAAAAACCATGGGTAGCATTAACCTGGCTATTGACG  CCCGAATTCAAAAGGAGGAAAAAACCATGGGTAGCATTAACCTGCGTGCTGACGA  TGAA  CCCCGAATTCAAAAGGAGGAAAAAACCATGGGTAGCATTAACCTGCGTATTGACG  ATGAACTTGCTGCGCGTTC  GATATACTCGAGCATGAGACGAAGCGCTTCAAGAGGAGTT  GATATACTCGAGCATGAGACGAAGCGCTTCACGAGGAGTT  CCCCGAATTCAAAAGGAGGAAAAAACCATGCGAAGGGCTTCTGTTGGTAGCATTA  ACCTGCGTATTGACG  CCCCGGATCCTCAACAGAGTTCATCCAGCGTCACACGTACTGG  CTCATGCTCGAGTATATCGC  ATGGGTAGCATTAACCTG  CAGGTTAATGCTACCCAT  TGTAGTGCGATACTTGTAATGAC  TACTTGTAATGACATTTGTAATTAC  GACATTTGTAATTACAAGAGGTG  ATTACAAGAGGTGTAAGACATGG  GTGTAAGACATGGGTAGCAT  ATGCTACCCATGTCTTACAC  TGTCTTACACCTCTTGTAATTAC  CTCTTGTAATTACAAATGTCATTAC  TACAAATGTCATTACAAGTATCG  ATTACAAGTATCGCACTACAAC  GGTGTCTGTACCAGTAAGATGAT  TGTCATTACACTTATCGCACTA  GTGCGATACTGTTAATGACATT  AATGTCATTAACAGTATCGCAC  GCGATACTTGGCATGACATTTG  CAAATGTCATGCCAAGTATCGC  GATACTTGTACGGACATTTGTA  TACAAATGTCCGTACAAGTATC  TACTTGTAATTCCATTTGTAATTACAAGAGG  CCTCTTGTAATTACAAATGGAATTACAAGTA  CTTGTAATGAACTTTGTAATTACA  TGTAATTACAAAGTTCATTACAAG  TGTAATGACAGGTGTAATTACAAG  CTTGTAATTACACCTGTCATTACA  TAATGACATTGTTAATTACAAGAG  CTCTTGTAATTAACAATGTCATTA  ATGACATTTGGCATTACAAGAGGT  ACCTCTTGTAATGCCAAATGTCAT  GACATTTGTACGTACAAGAGGTG  CACCTCTTGTACGTACAAATGTC  CATTTGTAATGCCAAGAGGTGT  ACACCTCTTGGCATTACAAATG  TTTGTAATTAACAGAGGTGTAAG  CTTACACCTCTGTTAATTACAAA  GACATTTGTAATTACACTAGGTG  CACCTAGTGTAATTACAAATGTC  TGTAATGACAGTTGTAATTACAAG  CTTGTAATTACAACTGTCATTACA  TGTAATGACATGTGTAATTACAAG  CTTGTAATTACACATGTCATTACA  TTTTTGTAATTACAAGAGGTGTAAG  AATTACAAAAATGTCATTACAAG  TTTTTTTTTTTTTGTAATTACAAGAGGTGTAAG  TTACAAAAAAAAAAAAATGTCATTACAAGTATC |

X denotes 5`-Cy5; nucleotide changes for site-directed mutagenesis in *relB* are underlined.

**Table S2.** Amino acid analysis of co-purified RelB-His_6_-RelE complex

| Amino acid^a^ | RelB  no. of residues | His_6_-RelE  no. of residues | RelB:His_6_-RelE 1:1 ratio | RelB:His_6_-RelE  2:1 ratio | Experimental value |
| --- | --- | --- | --- | --- | --- |
| Asx  Glx  Ser  Thr  Ala  Val  Met  Ile  Leu  Tyr  Phe  Lys  Arg  His^b^  Delta value^c^ | 9  11  4  3  6  5  2  4  13  2  1  5  7  0 | 5  12  7  1  4  12  1  5  10  5  3  11  10  6 | 14  23  11  4  10  17  3  9  23  7  4  16  17  6  77.3 | 23  34  15  7  16  22  5  13  36  9  5  21  24  6  32.9 | 23.2 ± 0.3  30.3 ± 0.3  19.3 ± 0.9  7.6 ± 0.4  25.8 ± 0.6  25.2 ± 0.3  5.3 ± 0.3  11.0 ± 0.6  36.7 ± 0.3  7.0 ± 0.1  4.2 ± 0.1  19.7 ± 0.3  20.0 ± 0.1  6.0 |

^a^No data was obtained for Pro, Cys and Trp, whereas data for Gly was omitted due to erroneous high values.

^b^His values for each experiment was used to normalize the measurement, because there are 6 histidine in the N-terminal tag of RelE and none in RelB.

^c^Delta values were calculated by taking the numerical sum of the experimental no. of residues subtracted from the theoretical values.

**Table S3**. Thermodynamic parameters of the thermal unfolding of RelB

| **[RelB] (μm)** | **pH** | **Tm (^o^c)** | **ΔHcal**  **(kcal/mol)** | **ΔH_v_**  **(kcal/mol)** | **ΔHcal/ΔHv** |
| --- | --- | --- | --- | --- | --- |
| 112 | 8.5 | 69.7 | 24.2 | 50.9 | 0.48 |
| 112 | 7.4 | 71.7 | 25.6 | 51.3 | 0.50 |
| 56 | 7.4 | 69.9 | 23.1 | 43.6 | 0.53 |
| 28 | 7.4 | 65.9 | 15.9 | 29.3 | 0.54 |
| 112 | 6.2 | 73.6 | 26.9 | 54.6 | 0.49 |
| 112 | 5.1 | 76.9 | 27.8 | 53.5 | 0.52 |

The thermodynamic parameters of the thermal unfolding of RelB were obtained from the model analysis (N_2_↔ 2U) of DSC endotherms. DCp = 0.4 kcal mol-1. Each experiment was repeated at least three times to estimate the relative error on the parameters by variation of the possible baseline positions.

**Legends to supplementary figures**

**Fig. S1. Analysis of the oligomeric state of RelB and RHH variants.** RelB and the mutant variants indicated (40 µM), were incubated in the absence (-) or presence of 0.5 mM (+) and 5 mM (++) BS^3^ crosslinking reagent at 22ºC for 60 min. Samples were quenched and subjected to SDS-PAGE. The gel was stained with Coomassie Blue to visualize protein bands. Molecular masses of the marker bands are indicated (left).

**Fig. S2. Deletion mapping of *relO.*** (a) A series of ^32^P-end-labelled DNA fragments truncated from -20 to +21 bp in decrements of 10 bp (indicated to the right) were incubated in the absence (-) or presence (+) of RelB•RelE complex (200 nM) and subjected to gel-shift assay and subsequent phosphoimaging. (b) A series of ^32^P-end-labelled DNA fragments truncated from +40 to -1 bp in decrements of 10 bp (indicated to the right) were treated as in (a)*.* The deduced minimum RelB•RelE binding site is marked in green on the lower bar.

**Fig. S3. Quantitative immunoblotting of RelB and RelE.** (a) Quantitative RelB and RelE immunoblotting. The cellular level of RelB as detected from a wild-type (MG1655) strain was compared to a dilution series of known amounts of purified RelB. (b) The cellular level of RelE was detected from a Δ*relBE* strain (MG1655Δ*relBE*) harboring pSC7104, a mini-R1 containing the *relBE* operon and its native promoter^8^. RelE levels were compared to a dilution series of known amounts of purified His_6_-RelE. From these and repeated experiments the number of RelB and RelE molecules (monomers) per cell are estimated to be in the range 550-1,100 and 50-100 for RelB and RelE, respectively.

**Figure S4. Thermodynamic properties of RelB, monitored by differential scanning calorimetry (DSC).** (a) Thermal unfolding of RelB (left panel) Two consecutive Cp versus temperature scans (pH 7.4) displaying a high degree of reversibility. (b) DSC thermograms of RelB measured at different protein concentrations, the change in heat capacity is expressed in kcal K^-1^ mol^-1^ of monomer. (c) Temperature dependence of ΔG^o^ at pH 7.4, for different concentrations of RelB.


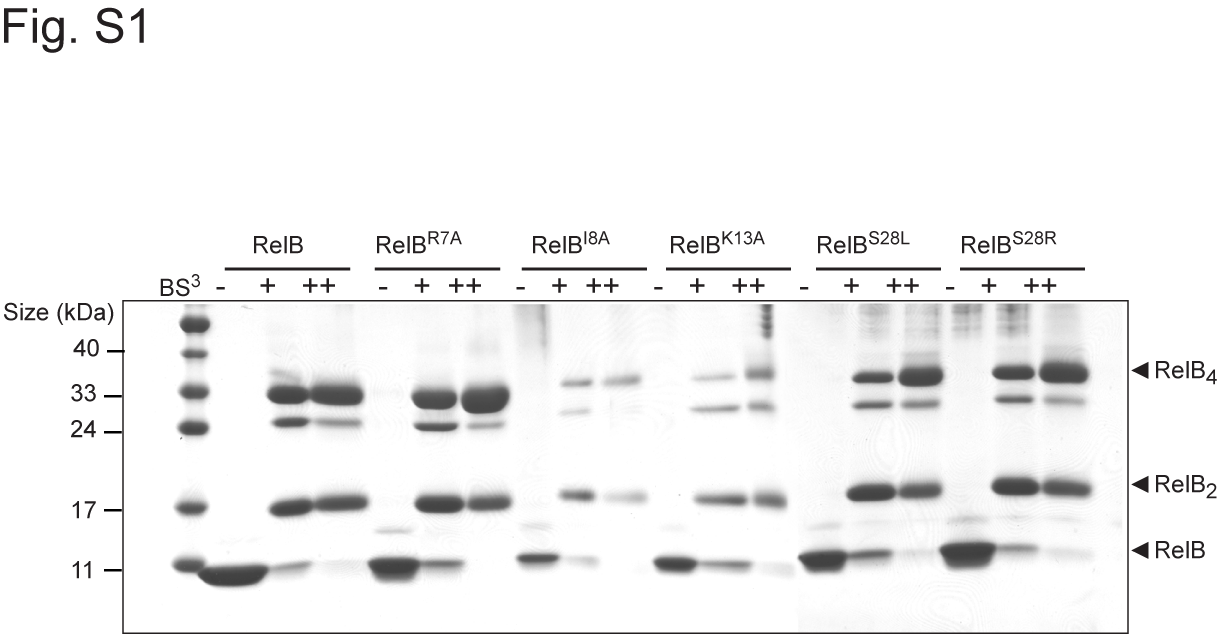


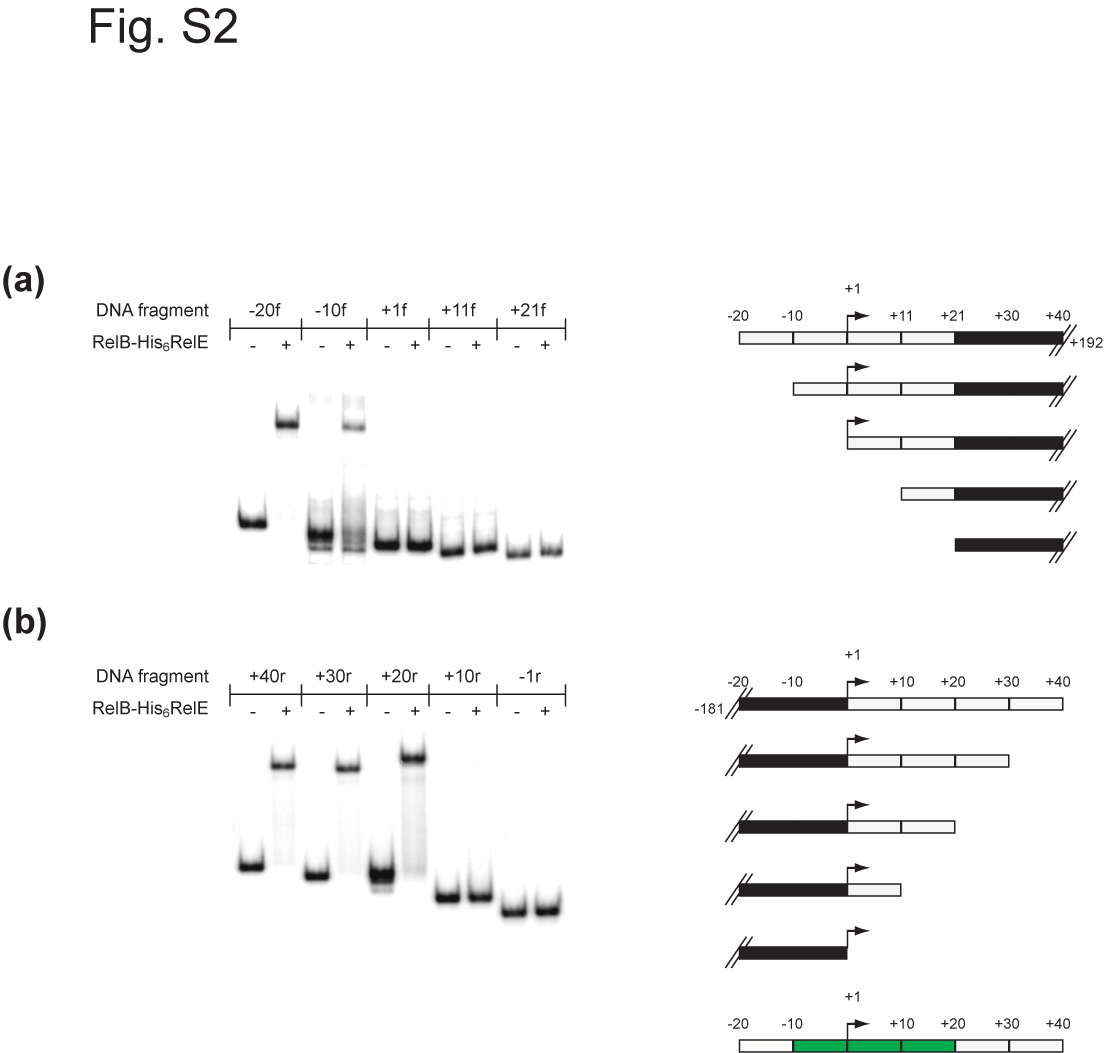


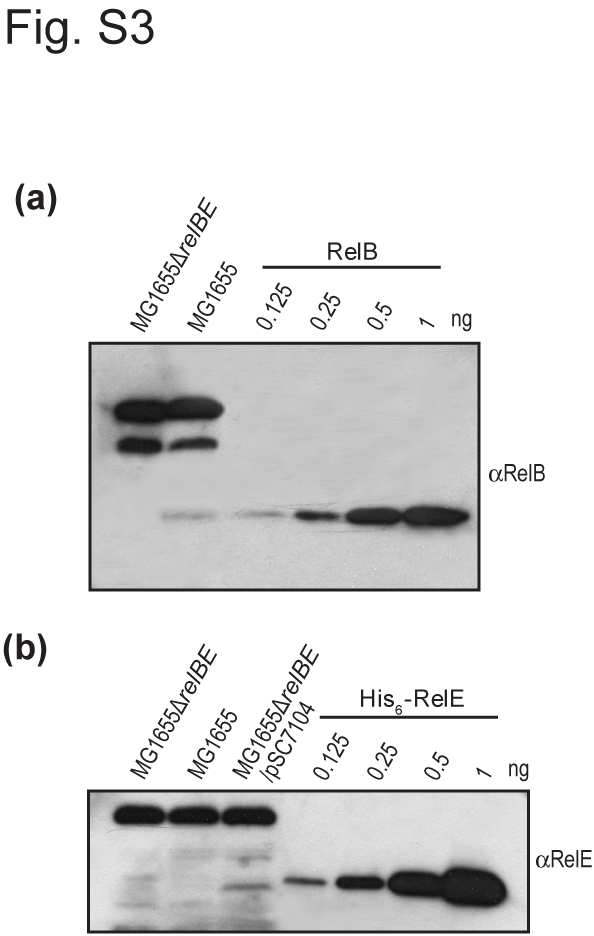


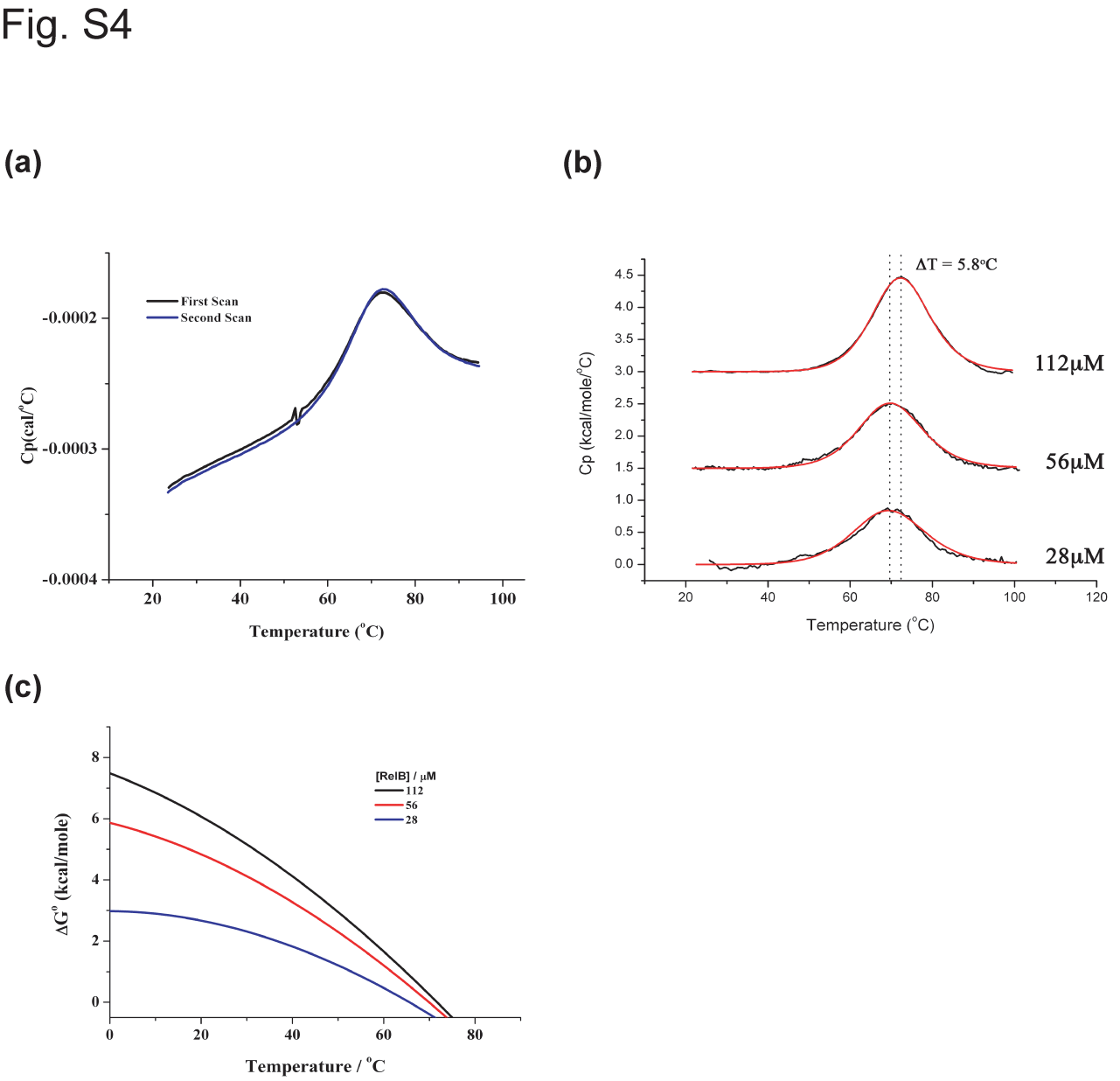

Supplement: Supplementary material [file mmc1.doc]
